# Robots for Elderly Care: Review, Multi-Criteria Optimization Model and Qualitative Case Study

**DOI:** 10.3390/healthcare11091286

**Published:** 2023-04-30

**Authors:** Bartosz Sawik, Sławomir Tobis, Ewa Baum, Aleksandra Suwalska, Sylwia Kropińska, Katarzyna Stachnik, Elena Pérez-Bernabeu, Marta Cildoz, Alba Agustin, Katarzyna Wieczorowska-Tobis

**Affiliations:** 1 Department of Business Informatics and Engineering Management, AGH University of Science and Technology, 30-059 Krakow, Poland; 2 Institute of Smart Cities, Department of Statistics, Computer Science and Mathematics, Public University of Navarre, 31006 Pamplona, Spain; 3 Haas School of Business, University of California at Berkeley, Berkeley, CA 94720, USA; 4 Occupational Therapy Unit, Chair of Geriatric Medicine and Gerontology, Poznan University of Medical Sciences, ul. Swiecickiego 6, 60-781 Poznan, Poland; 5 Department of Social Sciences and the Humanities, Poznan University of Medical Sciences, 60-806 Poznan, Poland; 6 Department of Mental Health, Chair of Psychiatry, Poznan University of Medical Sciences, ul. Szpitalna 27/33, 60-572 Poznan, Poland; 7 Geriatrics Unit, Chair of Palliative Medicine, Poznan University of Medical Sciences, os. Rusa 55, 61-245 Poznan, Poland; 8 Department of Applied Statistics and Operations Research, Universitat Politècnica de València, Plaza Ferrandiz y Carbonell, sn, 03801 Alcoy, Spain

**Keywords:** healthcare, elderly care, robotic assistant, service robot, social robot, human-robot interaction, caregiver, multi-criteria optimization, mathematical integer programming, artificial intelligence

## Abstract

This paper focuses on three areas: the first is a review of current knowledge about social and service robots for elderly care. The second is an optimization conceptual model aimed at maximizing the efficiency of assigning robots to serve the elderly. The proposed multi-criteria optimization model is the first one proposed in the area of optimization for robot assignment for the elderly with robot utilization level and caregiver stress level. The third is the findings of studies on the needs, requirements, and adoption of technology in elderly care. We consider the use of robots as a part of the ENRICHME project for long-term interaction and monitoring of older persons with mild cognitive impairment, to optimize their independence. Additionally, we performed focus group discussions (FGD) to collect opinions about robot-related requirements of the elderly and their caregivers. Four FDGs of six persons were organized: two comprising older adults, and two of the other formal and informal caregivers, based on a detailed script. The statements of older participants and their caregivers were consistent in several areas. The analysis revealed user characteristics, robot-related issues, functionality, and barriers to overcome before the deployment of the robot. An introduction of the robot must be thoroughly planned, include comprehensive pre-training, and take the ethical and practical issues into account. The involvement of future users in the customization of the robot is essential.

## 1. Introduction

During the recent decades, European societies have been aging, and this trend will continue. The increase in average life expectancy has reached a value of about three months per year. All age groups of 65 years and more have grown, with the fastest growth observed in the 80+ age cohort [1].

Undoubtedly, the older people are the ones who are most frequently in need of support regarding their daily activities. It should thus be a priority for the care systems and policies across the globe (in both the health and the social care sectors) to enable the older adults to live independently in their own homes, despite their possibly occurring physical and mental impairments, for as long as possible, therewith fulfilling the paradigm of aging in place [2]. The increasing numbers of older individuals in need of care, combined with limited availability of trained professionals in both the abovementioned care sectors, call for the implementation of new ideas and solutions aimed at reducing the (steadily increasing) burden on formal and informal caregivers. The involvement of the latter is considerable in many countries, for instance in the USA, Spain, Poland, Switzerland, Italy, and Greece [3]. The developed solutions must be both technically viable and economical.

In the case of application of robots for elderly care in the home, the usefulness of healthcare procedures is restricted by functional stagnation and a gap between the robotic platform and the intervention design [4]. To address this issue, researchers [4] suggest a novel co-design toolset for robot interactions in the healthcare area that employs an ecological paradigm. This strategy combines robot competences with known geriatric variables to generate a comprehensive view that includes both the physical platform and the deployment rationale [4]. Th human–robot relationship has grown increasingly prevalent, while robots have made everyone’s lives more convenient and easier [5].

As an example, social and service robots are designed to assist older people living at home [6], as well as in senior houses or hospitals. Among them, persons with mild cognitive impairment (MCI) constitute a separate group with distinct needs. MCI can be viewed as an intermediary state located between physiological aging and dementia [6]. This group might consist of the elderly, and there are subjects who get worse with time and may progress to dementia, as well as persons with sustained MCI in which the cognitive impairment stays at a constant level for many years, and also individuals who gradually improve. Care robots have an important role in helping this elderly group, since no reliable method of subjective distinction of the various prognoses of MCI exists; non-pharmacological interventions are the method of choice in individuals with MCI, with the aim of delaying their possible progression to dementia [6]. Robot are not only assisting seniors, but also supporting caregivers who provide help to elderly.

It is critical to gain a better understanding of how older adults perceive, think, and feel about the use of robots in aged care settings. For instance, the use of socially assistive robots (SARs) for older adults indicates distinct positive and negative attitudes toward various aspects of SARs in aged care. However, some opinions can be ambiguous and require further consideration if SARs are to be seriously considered for use in aged care. Understanding the lived experiences of older people with SARs allows for the use of an approach that incorporates technological advancement into the elderly care practice itself [7]. Socially assistive robots (SARs) have the potential to improve care delivery at home for seniors who have cognitive impairments, while also reducing the burden on informal caregivers. An interesting example is the field of SAR for dementia care (as mentioned above), which is expected to grow in the coming years. The importance of customizing the SAR appearance, services, and social capabilities are key factors for this area of application. Imbalances between needs and the robot’s solutions, usability issues, and a lack of prior experience with advanced technologies were identified as the most significant barriers to SAR adoption. Elderly people who are concerned about cognitive impairment recognize SAR’s potential to support health and social care at home. The current state of SAR research sometimes does not enable us to draw the conclusion that older adults are fully prepared for caregiving robots, but the concept is no longer unthinkable. However, many challenges must be overcome before SARs can be demonstrated to be effective, useful, and valuable enough to be employed as personal care assistants [8].

Socially assistive robots (SARs) have been identified as a potential solution to address the challenges associated with caring for the aging population. These robots are designed to interact with people and assist them in various ways, including providing social companionship, cognitive stimulation, and physical support. Studies have shown that SARs can help alleviate feelings of loneliness and depression in older adults, as well as improve their cognitive functioning and overall well-being [9].

Research has also shown that SARs can be effective in supporting individuals with dementia, reducing their agitation and improving their socialization with others. Additionally, SARs can assist with various physical tasks, such as helping with mobility and exercise routines, which can help prevent falls and other health issues [10].

Overall, SARs have the potential to significantly improve the quality of care for older adults and individuals with disabilities, while also alleviating some of the burden on caregivers. However, there are still many challenges that need to be addressed, such as ensuring that the robots are user-friendly, reliable, and can adapt to the needs and preferences of individual users. Moreover, ethical and privacy concerns must also be addressed to ensure that SARs are used in a safe and responsible manner [11].

In summary, SARs have the potential to play a significant role in care settings, providing support and companionship to older adults and individuals with disabilities, and reducing the burden on caregivers. While there are still challenges that need to be addressed, research suggests that SARs can have a positive impact on the physical, cognitive, and emotional well-being of users [11].

The presence of cognitive impairment has implications for care: it increases the demand and makes the provision more difficult. For example, older adults might need to be supervised in taking their medicines because they take their medications at the wrong time or forget entirely to take them—even if drugs have been placed in a clearly marked, dedicated container. If this happens, commonly, the caregivers (typically the family) control the medication-taking habits. Situations of this kind naturally stimulate the development of care innovations that utilize new technologies, comprising the introduction of socially assistive robots.

A clear preference of the majority of older adults is to age in place (i.e., where they have been living). However, with time, more and more of them are becoming dependent, which makes aging in place increasingly difficult. The difference between the abilities that are required for independent living and those one still has available to care for oneself is called the “care-gap”, which, traditionally, has been bridged with human care [12]. In present times, due to declining human resources and capacities [13], various robotics projects have emerged with the aim of assisting older persons [14]. Robots are expected to provide adequate support for both preventing functional decline and improving well-being [15]. However, assistive robots are only accepted if they are actually useful and effective for care [16]. These conditions are important in two ways: both because robots can be used to assist older persons in activities of daily living, and also because of their potential role as a factor which alters the social environment of the user’s home [17].

Gathering personal information and making use of it in care, while respecting the older persons’ choices, as well as employing their experience and subjective perceptions, are key issues of person-centered care. The development of a socially assistive robot should thus take into account the points of view of older adults as potential end-users [18]. Since the acceptability of the robot is linked to its functions and usefulness [19], if older people view the robot as useful, they are more likely to consider it acceptable. They have also been shown to be willing to accept assistive devices, both in healthcare and in housekeeping, to help maintain their independence, particularly when there is a perceived need for the device [20]. Additional determinants have been listed in the model of pre-implementation acceptance presented by Peek et al. [21]. The necessity for technology was only one of four descriptive factors here, along with social influence, the characteristics of older adults, and their perception of technology.

It was shown that the failure of some telemedicine projects could be at least partially attributed to a failure to properly assess the users’ needs [22]. An introduction of robots can face a similar scenario if no careful evaluation of needs and requirements is carried out. Proper initial assessment of needs and a feasibility analysis are essential for the success, and contribute to higher acceptance rates [23].

Results of studies concerning needs, requirements, and acceptance of technology for aging in place are still only rarely published in the medical literature [18,24]. One goal of the study is to convey the data acquired during focus group talks about the use of the robot in care for older persons—from both their and their caregivers’ perspectives.

Robots have been proposed as a potential solution to address the increasing demand for elderly care due to the aging population. However, the implementation of robots can be costly. An average implementation cost of a robot for elderly care in the US is approximately $85,000 per year. On the other hand, the cost of hiring human caregivers can also be high, as it involves not only salaries but also recruitment, training, and management costs. The average annual cost of a full-time caregiver in the US was around $75,000. Despite the high initial cost, robots may have long-term benefits as they can potentially reduce the need for multiple caregivers and provide 24/7 care, whereas human caregivers require breaks and time off. However, robots may not be able to provide the same level of emotional support as human caregivers, which may be crucial for elderly individuals.

Hence, the research flow consists of the following steps: we begin by reviewing the pertinent literature to identify potential synergies with existing literature. As a result, Section 2 includes a literature review, with the most important references on the subject. This includes problem contextualization, methodologies, and findings that may strengthen our work paper’s position. An overview of mobile service robot applications is in Section 3. In Section 4 a comparison of service robots versus social robots for elderly care is explained. The definition and design of a multiple-criteria conceptual optimization model is the following step, which can be shown in Section 5. A qualitative case is explained in Section 6. Finally, Section 7, Section 8, Section 9, Section 10 and Section 11 provide an analysis of the main findings and the computational results, as well as a discussion, limitations, future research directions and conclusions.

## 2. Review

A growing amount of research is being conducted to develop a human–robot interaction idea for service robots to support elderly people with physical activities at home. One particularly intriguing approach [13] in this area is founded on the recognition that robots are not yet capable of performing all tasks autonomously and reliably in the complex and heterogeneous conditions of people’s residences. While fully autonomous robots are not yet available on the market, this is an intriguing vision for providing functional support to the elderly in the near future. Similarly, in the construction of robots, machine learning methods have been utilized in a variety of sectors in conjunction with imbalanced approaches [25]. There is also a significant role for artificial intelligence (AI), a branch of computer science concerned with the development of intelligent machines, such as robots, capable of performing activities similar to those performed by people [26]. As technology advances, tasks currently performed by remote human operators may be gradually replaced by autonomous behavior and local engagement. In an ideal world, the proposed interaction design would be rendered obsolete when robots can satisfactorily manage all conditions independently [13].

The COVID-19 global epidemic has undoubtedly hampered access to health-care services around the world [27]. During that time, access to non-COVID health services was primarily limited and restricted around the world. The coronavirus—SARS-CoV-2—pandemic has had a maximal influence. Medical facilities around the world were unprepared for the obstacles posed by the increasing number of patients on a daily basis, and a lack of personal protective equipment as well as a shortage of medical staff. Health services have been disrupted in a variety of specialties, including primary care, psychiatry, orthopedics, cardiology, neurosurgery, and others [28]. Robotics integration in healthcare has gradually introduced effective tools for nurses and medical staff [29]. Due to a lack of powerful tools for assessing various factors in the formation of epidemic situations and forecasting, the SARS-CoV-2 coronavirus demonstrated inconsistencies in adequately responding to biological threats on a global scale. Considerations such as robot assistance in healthcare in general are critical, especially in the most fragile patients, such as adults. The use of accurate tools, as well as forecasting the evolution of new equipment applications in Medicare, is as important as forecasting of new cases and drug utilization, allowing for the testing of excess demand and tool demand to be managed more efficiently through the supply chain. During a pandemic, forecasting becomes critical for effective government decision-making, managing supply chain resources, and informing difficult policy decisions [30]. Not only does the COVID-19 pandemic highlight the need for novel solutions, such as the use of robots in elderly care, but (even more important) global demographics are pointing to an elderly society. As a result, there will be an upsurge in the social demand for care for the elderly. Assistive technologies, such as service robots, have recently emerged and can assist older adults in independent living. The attitude of older adults to using mobile service robots is encouraging, and such robots are assumed to play a significant role in aged care. However, there are some obstacles or unresolved issues to consider when designing mobile service robots. Because elderly users are in close contact with robots, their safety is critical. Furthermore, because robots monitor numerous environmental variables, they should ensure individual freedoms for elderly users by decreasing the numbers of sensors or providing clarity prior to use. Furthermore, ethical and cost considerations should be prioritized in the designing and developing [31]. 

According to recent research findings [32], older persons are reasonably open-minded about robots such as socially assistive robots (SARs), feeling at ease with at least some parts of them, and want to employ robots in the future, particularly for physical assistance activities. As a result, robots may be able to address some of the needs of the elderly. Although quantitative research aids the understanding of older individuals’ experiences with and views of robots, social contextualization is required to fully comprehend their meaning [33]. 

Robots may serve to promote social connection and minimize feelings of loneliness, increase medication compliance and adherence to health regimens, and increase independence [34], not only for the aged, but also for those with mild cognitive impairment (MCI) or Alzheimer’s disease and related dementias (ADRD), as well as to enhance caregiver well-being [35]. 

There are numerous applications for robots. Among them is the use of humanoid robots in Alzheimer’s disease and dementia care [36]. The authors investigated the acceptability of humanoid robots and the needs of users when using these robots to assist with care for people with Alzheimer’s disease and related dementias (ADRD); their family caregivers, health care professionals, and the general public are all affected. As a result of this study, significant relevance for the applications of social robotics in dementia care, as well as biomedical interventions related to artificial intelligence (AI) and robotics in healthcare, has been confirmed [36]. 

Another example of robots for elderly care is an anthropomorphic collaborative healthcare and domestic assistant robot capable of performing generic service tasks in non-standardized healthcare and domestic environment settings [37]. This robot can perform a wide range of chores, either independently or collaboratively. The main advantage of this robot is to provide a generic robotic solution such that older people can live longer, more independent, and healthier lives [37].

A different type of robot, which must be considered in this Section 2 is a pet robot. Affordable robot pets may have important well-being effects in older adults, including reduced neuropsychiatric symptoms (depression, delusions, elation, anxiety, and apathy), with qualitative accounts also supporting reductions in agitation. This type of robot usage impacts occupational disruptiveness, as an indicator of care provider burden [38]. Pet robots have been also employed as viable substitutes for pet therapy in nursing homes [39]. Pet robots are technology-based substitutes for animal-assisted therapy. Animal-assisted therapy has demonstrated positive benefits for the psychosocial well-being of people with dementia [40], such as reducing depression, providing companionship and addressing unmet needs [41]. However, using live animals can be challenging, due to issues such as logistical difficulties or potential transmission of zoonotic diseases [41]. Correspondingly, pet robots are considered as alternative solutions to circumvent such challenges, and have been used as non-pharmacological interventions to support the psychosocial health of people living with dementia [42]. There are several pet robots designed with varying levels of familiarity, reality and interactivity.

## 3. An Overview of Mobile Service Robot Applications

A service robot provides a variety of commercial and personal services to organizations and humans across a wide range of application domains. Nowadays, the service robot industry is expanding rapidly in tandem with the Fourth Industrial Revolution’s technological advances [43]. Robotics, a multidisciplinary field of computer science, computer engineering, ergonomics, artificial intelligence, organizational behavior, and mechanical engineering, is driving the phenomenal advancement of service robots [44]. As scientific communities push technological innovations in relevant fields such as artificial intelligence, hardware, and network technologies, the capabilities of service robots are expected to grow rapidly. COVID-19 is speeding up the demand for customer-facing service robots in numerous different companies such as hotels and healthcare services, in addition to the growth momentum driven by these technological innovations [45].

The expanding role of robotics in healthcare and related sectors has been investigated during and after the pandemic, with special emphasis on the management and control of the spread of the coronavirus disease [46]. There are numerous opportunities in the design and operation of medical robots, including a cyber-physical system (CPS), power saving using optimized algorithms and renewable sources, highly available control, and reliable designs for reliable and safe operation within outpatient clinics.

Even though mobile service robots work in close proximity to the elderly, it is critical to consider safety requirements [43]. In the context of human–robot interaction, a collision between the robot and an elderly person or a robot malfunction may occur. As a result, developers should broaden their understanding of mobile service robot safety issues, particularly in the context of elderly care. In general, for widespread acceptance, there should be a high level of trust between the elderly patients and the robot. As a result, when designing such robots, privacy and security must be prioritized [47]. It is difficult to trust new technology when older people are unaware of the amount of private data a mobile service robot is collecting or whether a third party is accessing their data.

### Robot Types on Different Robotic Platforms

In this paper, we selected 21 robot types on various robotic platforms as the most representative set of robot solutions available today.

These robot types are as follows:

- ARI [48]—is a mobile humanoid service robot whose primary design goals are to increase user acceptance of social robots and to use AI algorithms for caring.
- ASTRO [49]—assists elderly individuals with their indoor walking activities and physical training.
- Bandit [50]—is a wheeled platform with a humanoid torso. Physical exercise and cognitive training for the elderly are encouraged.
- Care-O-bot [51]—this robot can be used as a research platform as well as for a variety of tasks such as collection and delivery, independent living for the elderly, security or surveillance, and welcoming and guidance in retail stores or museums.
- Gymmy [52]—robot concentrates primarily on upper-body tasks.
- HealthBot [53]—robot can be used as a prescription or schedule reminder, a fall detector, an entertainment or memory assistant, and a phone caller. Furthermore, it monitors vital signs such as blood pressure, arterial stiffness, pulse rate, blood oxygen saturation, and blood glucose levels.
- Hobbit [54]—is a mobile platform with a depth camera for precise navigation and detection. A wireless call button, automatic voice recognition or gesture recognition interfaces, and a touchscreen allow users to engage with the robot.
- IRMA [55]—robot for locating misplaced objects that can be used to assist seniors.
- Kompaï [56]—a digital platform for social assistance. This robot promotes independent living and socialization among the elderly. The Kompaï robot performs a variety of functions, including day and night surveillance, mobility aid, fall detection, shopping list management, agenda, social connectivity, cognitive stimulation, and health monitoring. The robot can also recognize speech, navigate through unpredictable environments, avoid barriers, and detect potentially dangerous circumstances. Individuals connect with the robot using a touch screen and voice, and it has a little handle to assist the elderly in rising.
- Max [57]—a companion robot designed to provide long-term help to elderly persons at home.
- Pearl [58]—an autonomous mobile robot that responds to daily obstacles faced by the elderly, such as reminders and environmental direction. This robot can navigate autonomously, identify speech, distinguish faces, and compress photos in order to improve online video streaming with elderly relatives.
- Personal Robot 1 (PR1) [59]—this is a multifunctional human-assisting mobile manipulating robot developed to assist humans, particularly the elderly, in living independently and interacting with them.
- Personal Robot 2 (PR2) [60]—aside from a high level of engagement, like PR1, PR2 can see the environment in 3D, which aids it in autonomous navigation. Furthermore, it can walk dogs, fold clothing, open doors, and accomplish other similar chores, thanks to its flexible arms.
- PHAROS [60,61]—the platform includes human exercise recognition for exercise monitoring as well as a smart decision maker for recommending the most appropriate physical activities, based on the user’s physical limits.
- RAMCIP [62]—a service robot that offers safe and proactive everyday support to the elderly, particularly those with memory difficulties. This robot platform can be used for a wide range of tasks, including emergency detection (fall detection and gas/smoke detection), assistance in keeping the home safe (turning off electric appliances such as an oven or turning on lamps for locomotion), communication with relatives and friends, medication reminders, food preparation assistance, and picking up fallen objects.
- Robovie [63]—provides various services, such as helping with social isolation problems, daily greeting, conversing, assisting the elderly with complex activities, assisting in the grocery, and showing shop locations in a mall. This robot can communicate with humans by speaking and gesticulating, acting like a human youngster, and moving its eyes or head to express significant behaviors.
- Rudy [64]—using machine learning techniques, the robot provides remote monitoring, medication reminders, fall detection/prevention, and social networking.
- SCITOS A5 [65]—with a panoramic view, it is capable of people identification and tracking, object recognition, and 3D spatiotemporal mapping, and it provides entertaining programs for all ages. The robot’s frequency component for map improvement, which aids it in coping with environmental changes, is an exciting aspect. This robot was utilized as a walking companion for physical therapy groups of older people with advanced dementia.
- SoftBank Robotics Pepper [66]—robot was created for a variety of objectives, including cognitive training, health monitoring, companionship, scheduling reminders, greeting, discussion, surveying, educational purposes, entertainment, autism therapies, and even screening staff members during the COVID-19 epidemic. Using perception modules, the robot is capable of speech and emotional detection, sound localization, safe navigation, displaying body language, and engaging with the environment.
- Stevie [67]—robot is capable of providing long-term care for seniors and those with disabilities. This mobile platform has a human-like torso and two short arms.
- TIAGo [68]—a robotics research platform with basic environmental detection, learning, navigation, and obstacle-avoidance capabilities. Several research initiatives have made use of these robot platforms. The ENRICHME [69] project is one example of employing TIAGo to create a SAR for assisting the elderly, adjusting to their needs, and behaving naturally.

New robot types and platforms are constantly being developed, so more and more robots are being created, but we have chosen the most representative robots capable of elderly care for our review, based on our best knowledge.

## 4. Service Robots vs. Social Robots for Elderly Care

In this section, we explain the main distinctions, and complementary functions of both types of robots for elderly help and care: service vs. social.

Care robots come in a variety of sizes and designs. Some are intended for physical care, such as equipment that can help raise elderly people who are unable to get up on their own, assist with movement and exercise, monitor their physical activity and detect falls, feed them, and assist them in bathing or using the bathroom. A comprehensive comparison is explained as follows.

Service robots for elderly care are robotic equipment that help the elderly with their everyday routines and improve their overall quality of life. These robots can do everything from helping with domestic chores to offering companionship and monitoring health. Here are some instances of senior care service robots:

- Robotic Assistants: these are robots that can help with daily duties including cleaning, cooking, and medication reminders. They are outfitted with sensors and cameras to help them traverse the house and complete duties.
- Personal Robots: these robots are intended to offer elderly people companionship. They can converse, play games, and provide amusement. They are also equipped with sensors that detect falls and monitor vital signs.
- Telepresence Robots: these are robots that have video conferencing capabilities and can communicate with healthcare professionals, family members, and friends, remotely. They are also suitable for virtual tours and social gatherings.
- Robotic Exoskeletons: these are wearable robotic devices that can aid the elderly in their mobility. They provide aid and support during walking and can also be utilized for rehabilitation.

Overall, service robots for senior care are gaining popularity because they can improve the quality of life for the old while decreasing the workload on caregivers.

Social robots for elderly care are robotic devices that give the elderly emotional support and companionship. These robots are outfitted with sophisticated sensors, cameras, and microphones that allow them to perceive and respond to human emotions, movements, and facial expressions. Here are some instances of social robots used to assist the elderly:

- Companion Robots: these are robots that are developed to offer senior citizens companionship. They can converse, play games, and provide amusement. They are outfitted with sensors that detect and respond to human emotions, and they can also deliver medicine and appointment reminders.
- Pet Robots: these are robots that resemble the appearance and behavior of pets. They can offer the elderly comfort and emotional support if they are unable to have a live pet owing to physical constraints or living situations.
- Cognitive Assistants: these are robots developed to assist the elderly suffering from cognitive impairments such as dementia. They can serve as reminders for daily duties, aid with memory exercises, and provide cognitive stimulation.
- Telepresence Robots: these are robots that have video conferencing capabilities and can communicate with healthcare professionals, family members, and friends, remotely. They are also suitable for virtual tours and social gatherings.

Social robots for elderly care are gaining popularity, because they may provide emotional support and company to the elderly who are alone or lonely. They can also help to alleviate the strain on caregivers and doctors and nurses.

## 5. Optimization Model

The mathematical programing conceptual model can assist in the optimal assignment [70,71] of robots for elderly care. A triple-criteria optimization conceptual problem is designed to maximize the efficiency of assigning robots to assist older adults while minimizing stress, which is defined as a decrease in the efficiency of medical staff assisted by a robot. The model takes into account the need for robot assistance for older people as well as the minimization of workload for medical staff who are assisted by the robot. As a result of robot assistance, caregivers’ fatigue and stress levels are reduced. The purpose of this optimization model concept is to maximize the efficiency of assigning robots to serve the elderly. Nowadays, there are neither many publications about the utilization of mathematical programming models for elderly care, nor optimization models considering the relation between the minimization of caregiver stress and assignment of robots and other supporting tools in healthcare. Job stress encompasses not only work pressure but also time constraints and uncertainty. The healthcare literature recognizes that better work distribution among professionals diminishes stress levels and thus mitigates tiredness, which is a common factor in the health field, and potentially harmful to patients’ health care [72]. The deployment of robots to support caregivers may help to reduce the level of wrongdoing caused by tiredness [73].

### Multi-Criteria Optimization Robot Assignment for Elderly with Robot Utilization Level and Caregiver Stress Level (M-CORAEUS)

The importance of caregiver respite in the care of persons is extensively documented. Robotics has the potential to solve the demand for caregiver respite by promoting involvement and training environments for care recipients through ‘complementary caregiving’ activities. Robots may also provide caretakers with physical/emotional respite by giving companionship/friendship, as well as empowering individuals, safety/monitoring, and interactive participation [74].

It is critical to solve healthcare problems using mathematical programming methodologies and novel algorithms [75,76]. The use of multi-criteria optimization models as a decision-supporting tool for more efficient assignment of robots and caregivers for the elderly will become increasingly crucial as robots become more widespread in the future [77].

We have selected the following three criteria ($f_{i}$, for i=1…n, there n=3) in the multi(triple)-criteria weighted-sum approach optimization model: the maximization of efficiency in the assignment of robots/caregivers to the elderly, the maximization of the level of robot utilization, while serving the elderly and the minimization of caregiver stress level, while helping the elderly.

The non-dominated solution set of the multi-criteria mathematical programming model [78] can only be gained in part by the parametrization on λ of the weighted-sum program [79]:

Model $M_{\lambda }$

Maximization or minimization of $\sum_{k=1}^{m}\sum_{i=1}^{n}\lambda_{k}f_{i}$

where it is subject to some specific model constraints (as it is formulated in the model presented in this paper (Equations (1)–(9)), where $0\leq \lambda_{k}\leq 1,\;\;\forall \;k=1\ldots m;{\;\lambda }_{1}>\lambda_{2}>I>\lambda_{m};{\;\lambda }_{1}+\lambda_{2}+I+\lambda_{m}=1$; where $f_{i}$ is defined as criterion (objective) f in a multi-criteria objective function for a number of criteria from i=1 to i=n, and where in the M-CORAEUS model *n* = 3.

It is generally known, however, that even if the entire parametrization on λ is tried, the non-dominated solution set of a multi-criteria mathematical problem such as this cannot be fully ascertained (e.g., [80]). To find unsupported non-dominated solutions, some upper bounds on the objective functions should be added (e.g., [81]).

This integer (binary) programming optimization model is defined over the same set of nodes i ∈ I and j ∈ J, representing, respectively, the robots or caregivers and elderly people. Thus, the optimization model searches for the optimal assignment of care to older people and the optimal level of utilizations of robots, and finally the optimal level of stress of caregivers, with the objective of maximizing the total efficiency of the assignment of robots/caregivers to the elderly and the maximization of utilization of robots, while minimizing the level of stress of caregivers. The model decision variable, model parameters, and model criteria are described in detail in Table 1, Table 2, and Table 3, respectively.

Afterward, the mathematical programming model (M-CORAEUS) is defined as the following, with the multiple-criteria objective function (Equation (1)), which has the following mathematical formulation:

Maximize

(1) $$\;\lambda_{1}\underset{i\in I}{\sum }\underset{j\in J}{\sum }c_{ij}x_{ij}+\lambda_{2}\underset{i\in I}{\sum }\underset{j\in J}{\sum }a_{ij}x_{ij}-\lambda_{3}\underset{i\in I}{\sum }\underset{j\in J}{\sum }b_{ij}x_{ij}$$

subject to

(2) $$\underset{i\in I}{\sum }x_{ij}=z_{j},\;\forall j\in J$$

(3) $$\underset{i\in I}{\sum }x_{ij}=u_{j}+z_{j},\;\forall j\in J$$

(4) $$\underset{j\in J}{\sum }x_{ij}=y_{i},\;\forall i\in I$$

(5) $$z_{j}+u_{j}=1,\forall j\in J$$

(6) $$x_{ij\;}\in \left\{0,1\right\},\forall i\in I,\;\forall j\in J$$

(7) $$u_{j}\in \left\{0,1\right\},\forall j\in J$$

(8) $$y_{i}\in \left\{0,1\right\},\forall i\in I$$

(9) $$z_{j}\in \left\{0,1\right\},\forall j\in J$$

The multi-criteria model (M-CORAEUS) is expressed in Equation (1), compounded by the criteria described in Table 3, making Equation (2) and Equation (3) to Equation (9) define the constraints:

1. Equation (1) describes multi-criteria (triple-objective) objective function, where efficiency of robots/caregivers’ assignment and utilization of robots service level is maximized, while caregivers’ stress level is minimized.
2. Equations (2)–(4) describe the condition of the assignment considered for robots and caregivers for the elderly.
3. Equation (5) describes the condition that assigns either caregiver or robot to the elderly.
4. Equations (6)–(9) describe the variable ranges.

Computational experiments with the use of only experimental data were performed using the AMPL programming language and the Gurobi 9.0.2 solver on a MacBookAir laptop with Dual-Core Intel Core i7 processor running at 1.7 GHz and with 8 GB RAM. The size of the integer (binary) program for the example problem was relatively small. The set of non-dominated optimal solutions was obtained within seconds. Due to the lack of access to real data for computations, the above mathematical programming multi-criteria optimization model (M-CORAEUS) was verified with the use of experimental data. The size of the experimental data set was the following: we considered 100 robots and caregivers and how they were going to be assigned for assisting/helping 100 elderly. Authors name this model conceptual. For this reason of experimental data use only, even the proposed set of mathematical equations gives promising solutions. This part of the research is going to be continued in the future as a next stage of this project.

Based on the best knowledge of the authors and after a careful check of published research papers, this proposed multi-criteria optimization model M-CORAEUS is the first one proposed in the area of optimization for robot assignment for the elderly with robos utilization level and caregiver stress level.

## 6. Materials and Methods

This research was conducted as part of the ENRICHME project (*ENabling Robot and assisted living environment for Independent Care and Health Monitoring of the Elderly*), aimed at robotic support of older persons with MCI. The project was approved by the Bioethical Committee of the Poznan University of Medical Sciences, Poland. The collection of potential end-users’ opinions about older persons’ robot-related needs and requirements was part of the project. We previously presented the findings of quantitative experiments that used the Users’ Needs, Requirements, and Abilities Questionnaire (UNRAQ)—a mixed-methodology tool developed as a component of the research project [82,83,84,85,86,87,88,89,90,91,92,93,94].

One of this research goals is to discuss the qualitative data stemming from focus group discussions in which potential users of the robot (i.e., older participants and their caregivers) were involved. The method of focus group discussions is predominantly recommended in those research activities where it is important to gain insights into the behavior and the motivations of participants and how their opinions are formed. It is also used to determine factors by which participants make decisions, and the hierarchy of values in their lives. Group processes make the focus group method particularly useful for investigating and recreating the conceptual system of the users and for elucidating their way of thinking about the studied issues. Therefore, we have selected this method as adequate for exploring and assessing the potential long-term impact of a social service robot in the interaction and monitoring of its human users, aimed at helping the older persons to stay active and independent as long as possible [95].

Focus group interviews in our study were based on a detailed script which had been prepared solely for this purpose. All participants gave their consent to participate in the study and to the recording of their opinions. At the beginning of each focus group discussion, short videos delivered by Robosoft (France), were presented, showing the robot to be used at the users’ homes. The main part of the discussion concentrated on two vignettes (named Maria and Jan). Both of these stories were prepared by a group of specialists involving a geriatrician, a psychiatrist, and a psychologist from the Poznan University of Medical Sciences, taking into account their expertise in the clinical picture of MCI. The proposed cases were debated and agreed on by all partners of the ENRICHME consortium.

Four focus group discussions were organized, with six persons participating in each of them: two groups of older adults and two of other stakeholders (one of the formal and one of the informal caregivers).

Focus group discussions with older individuals were arranged at day centers, which offer opportunities to participate in various activities, including organized trips as well as sessions of occupational therapy, art therapy, physiotherapy, and psychotherapy. The majority of the older persons were independent enough to reach the day centers on their own. The participants in the focus group interviews are presented in Table 4. They were eager to discuss the introduction of a robot and all issues related to its use. During one of the discussions, three students and their teacher were additionally present as observers (one pharmacy student from the English-speaking program of the Poznan University of Medical Sciences, and two visiting students from the College of Pharmacy, University of Kentucky, USA).

### Data Analysis

The focus group discussions were recorded and subsequently transcribed. The transcripts were analyzed based on the grounded theory methodology described by Strauss and Corbin [96,97,98]. One of the main advantages of this approach lies in its well-structured and practice-oriented method in generating grounded theory [99]. A key issue in this approach is coding—the operation by which data are broken, conceptualized, and put back together in new ways. The open coding, and, subsequently, the axial and selective coding, was performed independently by two researchers. The discrepancies were discussed until a consensus was found.

## 7. Results

The participants were engaged in the discussions and willing to express their thoughts. The statements of all studied groups were consistent in several areas. In total, 964 items were identified in open coding (some of them occurred more than once), which were subsequently assigned to subcategories in four main categories (Table 5). The participants discussed:

- WHO is the potential beneficiary of owning/using a robot? What distinguishes the robots’ users from other older adults?
- WHAT should the robot be like? Which features are indicated as meaningful?
- WHAT could the robot be used FOR? Which are its most important functions in terms of its usability?
- HOW should the robot be implemented into the care provision for older individuals? Which potential problems must be solved for the robot’s introduction to be effective?

**Figure 1:**
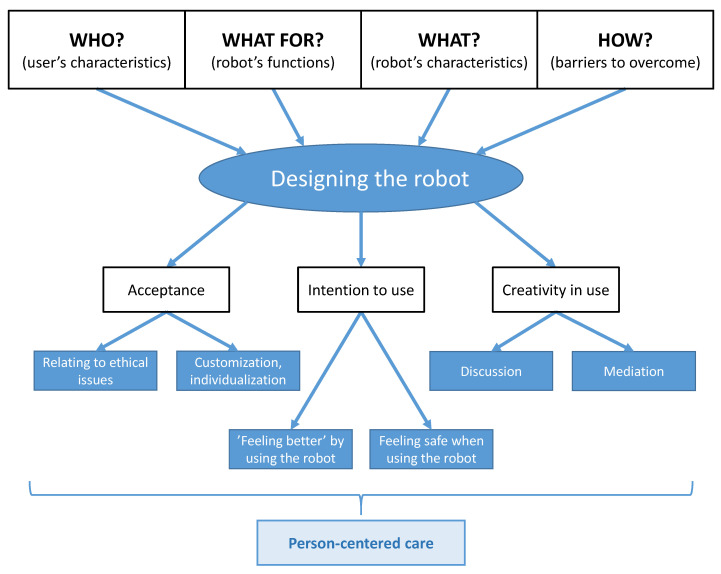
The identified categories.

After the analysis of the transcripts, a diagram was developed to present the identified categories (Figure 1). There are four key items which converge in the process of designing the robot: it must be customizable and deployed in an individualized way (WHAT), and also meet the unique expectations (WHO) regarding the robot’s functions (WHAT FOR) and the way it is introduced (HOW). A proper design is a crucial condition for the robot to be accepted by its user.

During the discussion, the following were pointed out:

1. The participants on the one hand stressed that the robot is “*a machine that has no emotions (...) and human (...) does not know all of the machine’s behavior; (...) I do not know if it would be nice for older people; I understand that younger ones have a different view because they are more familiar*” (A). On the other hand, it was stated that the robot is *“(…) a friend for all that matters, universal”* (3), and *“a friend because a friend always gives good advice (…)”* (2).
2. It should be emphasized that older participants had a lot more expectations towards the robot than the caregivers, and these expectations were more elaborated, for example:
3. Conducting discussions—participants pondered if “*it would be possible to talk politics? (…) But, what if the robot had different political views?*” (8) “*I could have an opponent. The discussion would be better then*” (9).
  - Safety issues—they discussed the possibility of the opening of the door by the robot and checking who comes in, with the decision not to let people come in to cheat/rob the older person. There appeared a question, *“would it let a thief in?”* (6) and an observation, *“someone who has been let in could damage the robot.”* (8)
  - Mediation—the robot could (as an impartial party) participate in solving conflicts with neighbors or family, and everybody would adhere to its decisions, *“There are problems with your wife or with children, and you can ask the robot to solve the problem.”* (6)
4. The participants agreed that *“for sure, the robot will not replace a human but, (…) if someone wanted that, let them cooperate [play or work together].”* (B) In this context, we found further statements, notably: *“a human always looks in a different way because he has sight because he has eyes, facial expressions, and feelings,”* and (F) *“this is simply equipment, and a man needs another man.”* (5) Thus, both the older persons and the caregivers clearly distinguished the presence of the robot from that of a human.
5. It was pointed out that robots are a solution for the future. The robot *“is a good thing, only that we will not wait.”* (5) *“I think it will work fantastic for people who are now 15 years old, as they will be 70–80; this will be the ideal solution.”* (H)
6. Much of the talk was devoted to ethical issues. Among other topics, it was discussed who should have control over the robot and access to the observational data, *“I cannot imagine the robot would be programmed so that my son or daughter could control it and that it would follow their orders (...), I would feel incapacitated”* (1). It was clear to all participants that access to data must be limited and that *“not that one will come and see, only the one person authorized to do so;”* (1) for example, if it was a daughter, *“the mother would have to agree to the daughter’s right for insight.”* (1) To the formal caregivers, it was apparent that, while the monitored health parameters could be transmitted, observing the older user (image transmission) is *“entering with shoes in [violating] someone’s privacy.”* (I). The potential users expressed the wish not to be spied on by the robot. Hence, the robot’s functions must be considered and implemented in an individualized way, to avoid the sense of “being controlled” from the perception of the older robot’s user.

The resulting theory puts the older user of the robot at the center of all related activities. It would have been all but wrong to confront older persons with machines designed by their younger counterparts with the just state of the art of the technology in mind. Older adults, when expressing their thoughts about the potential use of robots as their caregivers, reveal a wealth of reflections, and formulate statements from which precise hints for the designers can be drawn. The emphasis on the robot’s older users, and their needs and preferences, can be viewed as a practical emanation of the paradigm of person-centered care in the era of artificial intelligence and autonomous systems.

### The Mathematical Programming Conceptual Optimization Model—Findings from Computational Experiments

**Figure 2:**
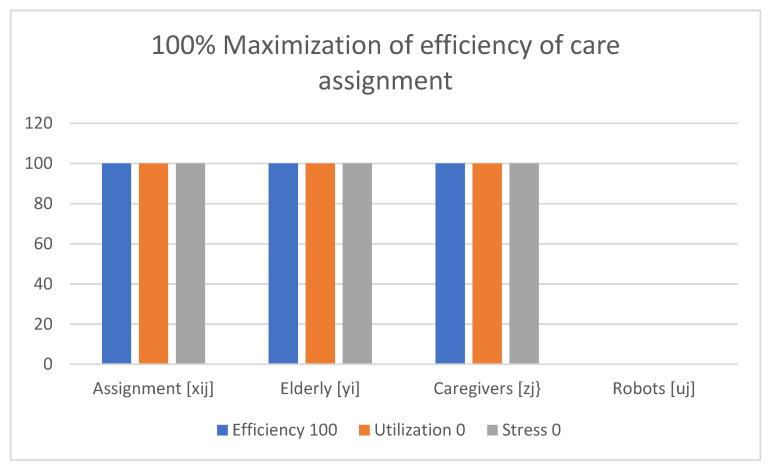
Example of solutions obtained with use of M-CORAEUS mathematical model for considered criterion of 100% maximization of efficiency of care assignment.

**Figure 3:**
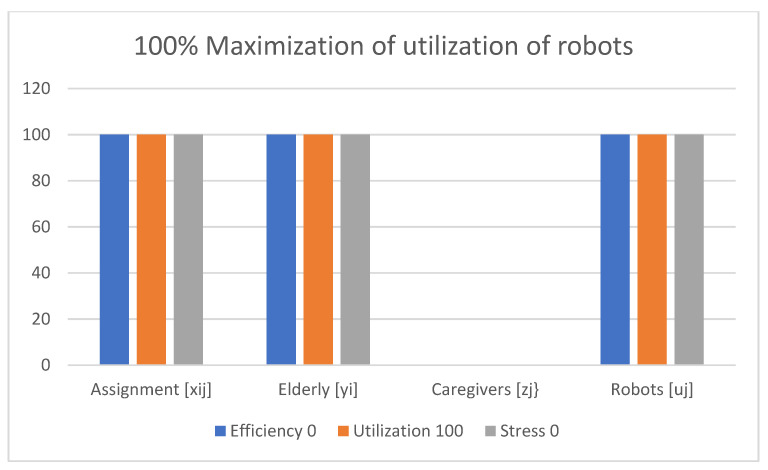
Example of solutions obtained with use of M-CORAEUS mathematical model for considered criterion of 100% maximization of utilization of robots.

**Figure 4:**
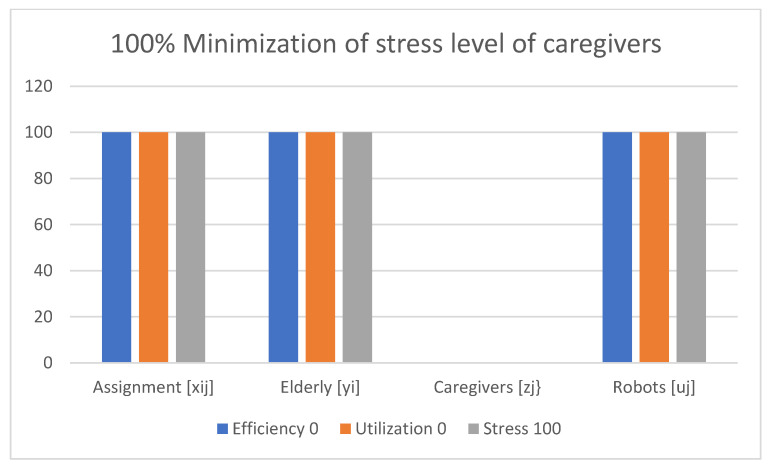
Example of solutions obtained with use of M-CORAEUS mathematical model for considered criterion of 100% minimization of stress level of caregivers.

**Figure 5:**
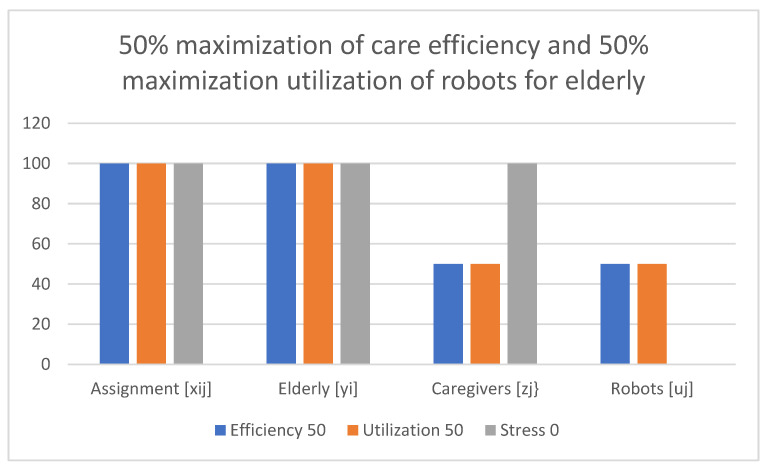
Example of solutions obtained with use of M-CORAEUS mathematical model for considered criterion of 50% maximization of care assignment efficiency and 50% maximization of utilization of robots.

**Figure 6:**
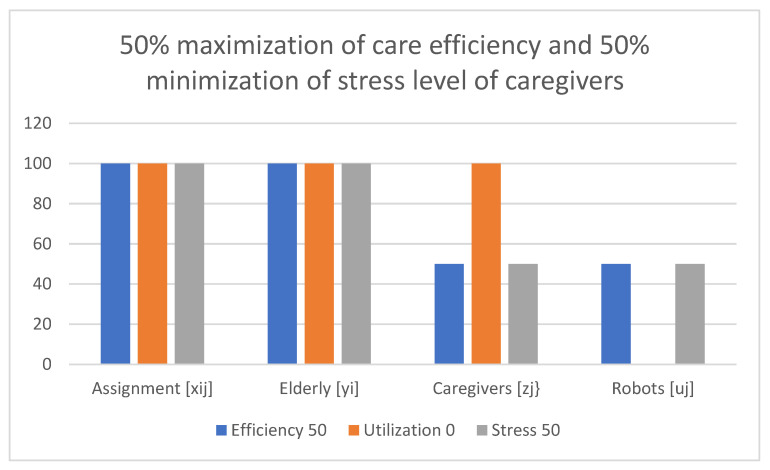
Example of solutions obtained with use of M-CORAEUS mathematical model for considered criterion of 50% maximization of care assignment efficiency and 50% minimization of stress level of caregivers.

**Figure 7:**
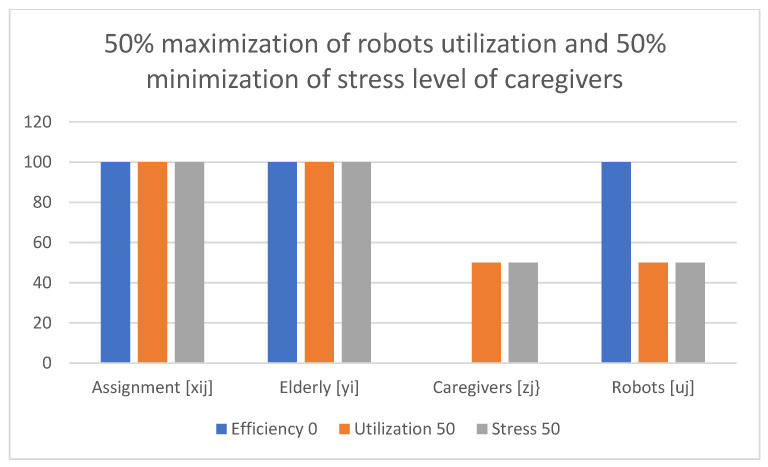
Example of solutions obtained with use of M-CORAEUS mathematical model for considered criterion of 50% maximization of robot utilization and 50% minimization of stress level of caregivers.

Examples of obtained results with the use of the multi-criteria optimization model for robot assignment for the elderly with robot utilization level and caregiver stress level (M-CORAEUS) are presented in Figure 2, Figure 3, Figure 4, Figure 5, Figure 6 and Figure 7. An experimental dataset was considered, to check the efficiency of the formulation of the conceptual model. We tested and solved the model using only experimental data; thus, we call it “conceptual”, yet the model is capable of finding a non-dominated set of Pareto optimal solutions.

The presented results of the assignment of robots and caregivers for the elderly in Figure 2 show the example of solutions obtained with the use of the M-CORAEUS mathematical model, when the considered criterion is 100% maximization of efficiency of the care assignment. In this case, only the caregivers (real people) are assigned to help seniors.

The presented results of the assignment of robots and caregivers for the elderly in Figure 3 show the example of solutions obtained with the use of the M-CORAEUS mathematical model, when the considered criterion is 100% maximization of utilization of the robots. In this case, only robots are assigned to help seniors.

The presented results of the assignment of robots and caregivers for the elderly in Figure 4 show the example of solutions obtained with the use of the M-CORAEUS mathematical model, when the considered criterion is 100% minimization of the stress level of the caregivers. In this case also, only robots are assigned to help seniors.

The presented results of the assignment of robots and caregivers for the elderly in Figure 5 show the example of solutions obtained with the use of the M-CORAEUS mathematical model, when the considered criterion is 50% maximization of the care assignment efficiency and 50% maximization of the utilization of robots. In this case, assignment is evenly divided between caregivers and robots.

The presented results of the assignment of robots and caregivers for the elderly in Figure 6 show the example of solutions obtained with the use of the M-CORAEUS mathematical model, when the considered criterion is 50% maximization of care assignment efficiency and 50% minimization of the stress level of caregivers. In this case, assignment is also fairly divided between caregivers and robots.

The presented results of the assignment of robots and caregivers for the elderly in Figure 7 show the example of solutions obtained with the use of the M-CORAEUS mathematical model, when the considered criterion is 50% maximization of robot utilization and 50% minimization of the stress level of caregivers. In this case, assignment is evenly divided between caregivers and robots, too.

In all six presented in this section of the paper (see Figure 2, Figure 3, Figure 4, Figure 5, Figure 6 and Figure 7) extreme cases (in terms of each of the three criteria values), 100% of the elderly were always assisted by caregivers or robots.

Computations were carried out using a MacBookAir laptop with a Dual-Core Intel Core i7 CPU running at 1.7 GHz and 8 GB RAM, using the AMPL programming language and the Gurobi 9.0.2 solver. For the sample problem, the integer (binary) program proved to be small in size. Within seconds, a set of non-dominated optimal solutions was generated. Due to a shortage of real-world data for computations, the aforementioned mathematical programming multi-criteria optimization model (M-CORAEUS) was validated using experimental data. The following is the size of the experimental data set: we consider 100 robots and caregivers, and how they are going to be assigned for assisting/helping 100 elderly. The authors refer to this model as conceptual. Because only experimental data is used, even the recommended set of mathematical equations provides promising solutions. This research will be continued in the future as part of the project’s next stage.

## 8. Discussion

Even though we observed some skeptical opinions related to the use of robots (older people frequently have worries about new technologies that may be challenging to comprehend) [19], its broad acceptance among the older people must be stressed. Nonetheless, at the same time, they stated that a user-friendly early introduction stage was indispensable. In this phase, conditions should be created that enable the older user to become familiar with the robot and gradually acquire confidence in their ability to operate it. Only after the older person gains proficiency in the operation of the robot, can the involvement of the personnel diminish—albeit flexibly, according to the level of self-reliance gained by the user. One may recall a statement in the European Digital Agenda for Europe: *“the more Europeans know [robots], the more they like them”* [100].

### Four Categories from the Focus Groups

Our focus groups (see Table 5) indicated wide acceptance of all suggested robot functions. Fairly high acceptance from both older adults and their caregivers was also observed in the study of Mast et al., who found that there was considerable demand expressed by both older adults themselves and by younger persons from their environment, for robots providing broad support [13]. Notably, older individuals in our study did not voice the opinion that the robot should perform everyday chores (instead of themselves), but they tended to report expectations regarding their training and support for their own activity. Thus, they perceived the robot rather as a stimulus than an apparatus taking over their daily activities. Our participants expressed the opinion that a person *“being served”* would *“sit down and never get up.”* Such robotic implementations may, in the longer term, erode the quality of life of older persons. A robot doing too much can harm the skills and motivations, or otherwise weaken the capabilities of the older user [8]. The robot in such a case would not be a helper, but rather a “wedge” that makes the care gap more severe. An intervention using a robot may also have nonspecific social stimulation effects (by inducing and strengthening social contacts), as well as more specific effects concerning cognitive training and physical activity. It is vital to take these issues into account when elaborating on the robot’s functions and designing its interface with the human.

## 9. Limitations

According to all our participants, the robot can have many real uses for older persons; however, all groups did mention some barriers to it. The recommendations they expressed should be taken into consideration during the development of the robot, although some of the points are not realistic within the current state of technology. Based on the analysis of the focus group discussions, we formulated four detailed questions (presented in the Section 2) to facilitate the development process. The robot’s designer needs to know its target group of users, with all their expectations and requirements, to be able to make the robot comply with them. As every person is unique, so are their wishes towards the robot. The robot must thus be customizable, and its design should provide appropriate configuration options. Demand for an individualized robot was clearly present in all discussions with both older persons and caregivers. An “individualized” robot was understood as having precisely these functions, which are essential for the user; all other functions should be eliminated or disabled, as they would make the robot’s operation unnecessarily complicated. The design should be functionally complete and easy to use, taking into account the expertise of various professionals [101]. Robots need to be tailored particularly to people who may be skeptical or reluctant to use them [19].

An important strength of the focus group method is the collection of opinions of several individuals and insights gained into the way they are formed, including the group processes and mutual stimulation. The inclusion criteria were not particularly specific; hence, no gender balance was maintained: the groups of older persons comprised four males (out of twelve) and the groups of caregivers—two males (out of twelve). This gender disproportion, however, reflects the male/female ratios in the oldest age cohorts, and also in both formal- and informal-caregiver groups in Poland and other European countries.

Another limitation of our study is the number of participants; despite that, we were able to engage representatives of all possible stakeholders in the discussions, as it is important to reflect various perspectives [14]. Kachouie et al. specifically pointed out that only a few studies included caregivers other than the older persons [18].

Person-centered and tailored care presents a challenge for research and contemporary medicine, particularly so in the wake of a growing demand for care [102]; the fact that it is being given inadequate attention in the context of robotics is stressed in the literature [18]. A tailored support package may include a robot for care, as it provides options for considering not only the individual’s health and social situation but also their interests, hobbies, and daily activities. Making use of personal opinions in care, viewing the older individual as a mature person [103], as well as respecting their choices and taking into account their previous experiences [104], are essential for person-centered care.

The categories and subcategories we derived from the analysis of the discussions using the grounded theory methodology are coherent with the principles of person-centered care. The *user’s* and *robot’s characteristics* put the potential end-users and their views at the center, relating both to the mental and the physical aspects of the human–-machine interaction. In addition, the *robot’s functions* are discussed from the users’ perspective, with their functioning, independence, and (daily) training in mind. Eventually, the *barriers to overcome* clearly concentrate on the user’s willingness to accept the robot and share their life with it, also taking into account their ability to operate the robot, as well as a range of ethical considerations [105,106].

## 10. Future Research

Future research in this area could go in three directions. The first step is to create new robots and procedures for improving human–robot interaction. The second is the use of multi-criteria mathematical programming models [107,108] with a simulation-optimization approach [109] to improve the level of support provided by robots to the elderly and caregivers, as well as the optimal placement of robots in eldercare. The third direction is closely tied to the difficulty of teaching robots how to interact with humans.

## 11. Conclusions

Wide-ranging interest concerning the use of robots in many aspects of care for older people living in their homes, senior houses and other medical facilities was observed during focus group discussions. Both studied groups (older persons and their caregivers) expressed significant demand for robots providing broad support. Special attention must be paid to the procedure of the robot’s introduction: it should be preceded by a comprehensive pre-training, and taking into account a range of ethical and practical issues. It is vital to involve the future robot’s users in the preparation and customization of technological solutions to be introduced, following the actual needs and preferences of the older people. Both the technical environment of the robot and its functions must match the user’s profile, including their priorities and the need for independence. Although robotics technology is developing, its widespread usage in the home, hospital, and care settings may be a long way off [110].

In this complex research paper about elderly care with the support of robots, we not only provide details about qualitative case studies, but also present a review of robots for older people together with a multi-criteria conceptual optimization model for robot assignment for the elderly with robot utilization level and caregiver stress level, based on mathematical integer (binary) programing.

Our ultimate goal was to provide not only a review of the state of research on the use of robots for eldercare, but also the findings of empirical studies and a mathematical model that would optimize robot-related problems. The model is a decision-making tool which illustrates how to use robots in such a way that they not only aid the elderly but also support caregivers.

## Figures and Tables

**Table 1:** Model decision variables.

Decision Variable	Description
***xij***	1 if Robot/Caregiver *j ∈ J* is assigned to Elderly *i ∈ I*, 0 otherwise
***yi***	1 if Elderly is considered *i ∈ I*, 0 otherwise
***zj***	1 if Caregiver is considered *j ∈ J*, 0 otherwise
***uj***	1 if Robot is considered *j ∈ J*, 0 otherwise

**Table 2:** Model parameters.

Parameter	Description
$\;\lambda_{k}$	Weight for criterion *k ∈ K* in the multi-criteria objective function
***c_ij_***	Assignment efficiency for robot/caregiver *j ∈ J* to elderly *i ∈ I*
***a_ij_***	Level of robot *j ∈ J* utilization, while serving elderly *i ∈ I*
***b_ij_***	Caregiver *j ∈ J* stress level, while helping elderly *j ∈ J*

**Table 3:** Efficiency, utilization, and stress criteria included in the multi-objective function.

Criterion	Description
$\sum_{i\in I}\sum_{j\in J}c_{ij}x_{ij}$	Efficiency of assignment of all robots/caregivers to all elderly
$\sum_{i\in I}\sum_{j\in J}a_{ij}x_{ij}$	Level of all robots’ utilization, while serving all elderly
$\sum_{i\in I}\sum_{j\in J}b_{ij}x_{ij}$	All caregivers’ stress level, while helping all elderly

**Table 4:** Characteristics of the participants of focus group discussions.

Focus Group		Age (Years) /Sex	Former Profession	Focus Group		Age (Years) /Sex	Role
**65+** **#1**	1	78 (F)	Shop-assistant	**Informal caregivers**	A	51 (F)	Caring for a family member
	2	77 (F)	Administrative employee		B	21 (F)	
	3	82 (M)	Engineer—designer		C	21 (F)	
	4	68 (F)	Tailor		D	21 (F)	
	5	86 (M)	Bookbinder		E	69 (F)	
	6	73 (F)	Graphic designer		F	70 (M)	
**65+** **#2**	7	76 (F)	Biochemist	**Formal caregivers**	G	41 (F)	Physiotherapist
	8	87 (F)	Office employee		H	52 (F)	Nurse
	9	77 (F)	Government employee		I	53 (M)	Art therapist
	10	79 (F)	Surveyor technician		J	32 (F)	Psychologist
	11	66 (M)	Company CEO		K	51 (F)	Social worker
	12	70 (M)	Postman		L	45 (F)	Nurse

**Table 5:** Summary of categories and subcategories identified during focus group discussions related to robots in the care for older people.

Category	Subcategory	Examples	Citations from the Discussions of Older Persons
**User’s characteristics**	Psychosocial issues	Loneliness, companionship, ability to operate the robot	The robot would give *“a sense of security when one is lonely (...) because I would like to wake up thinking that one is already there; one would feel less lonely with a robot”* (1)
	Medical issues	Multimorbidity, cognitive impairment, disabilities	*“He has illnesses, diabetes and hypertension, and he does not remember much and he is upset about it, so this robot would be very much needed by him”* (8)
**Robot’s characteristics**	Appearance	Humanoid or machine-like, head/face, skin/fur (tactile features)	*“I think it is good that it does not resemble a human being, that it actually looks like a machine, because if it had limbs, even immobile ones, it would be scary”* (B) *“I was thinking about some fur, the robot can be rendered more humanoid, dressed, decorated”* (H)
	Capabilities	Customizable, individualized for the user	*“It must not be a blabber, after all, it is there to keep the secret, not to betray to outside”* (2)
**Robot’s functions**	Assistive functions	Home safety, housekeeping, food preparation, being informative, help with reading, praying together	*“When it comes to cleaning, [the robot] cleans only in the middle, not in the corners.” “It is not able because it’s a manual thing, to bend over and yet make an effort”* (9)
	Health-related functions	Reminders (medications, doctor appointments), monitoring of life parameters, keeping medical records, physical exercises, cognitive games	*“If you faint and the ambulance comes, it could tell the doctor what is wrong with you, it could have your medical history inside”* (11)
	Social functions	Contact with the outside world, entertainment (playing cards, music replay)	*“I would like it to read some books, a chapter every other day, because everyone has his eyes tired”* (9)
**Barriers to overcome**	Ethical issues	Control over the robot, access to observational data, the right to disobey the user’s command	*“If someone sponsored such a robot to me, I would have a feeling that I was under control”* (11)
	Fears	High price tag, the risk of breakdown, loss of abilities if routine jobs are performed by the robot	The robot “*will do what one does not want to do, whether one is sleepy, one wants to rest, and it will just be doing other things and disturbing*” (6)
	Introduction	Gradual, staged introduction, pace of introduction matching the user’s capabilities, presence of a human assistant on-site (as long as necessary)	At the beginning *“there would have to be another person there because I would be afraid to remain with it alone”* (6), *“to learn how to live together”* (2)

## Data Availability

Data available on request from the authors.

## Reporting

When reporting our findings we observed the Standards for Reporting Qualitative Research (SRQR) [111].
